# Prediction of potential disease-associated microRNAs by composite network based inference

**DOI:** 10.1038/s41598-018-34180-6

**Published:** 2018-10-25

**Authors:** Bin-Sheng He, Jia Qu, Min Chen

**Affiliations:** 1 The First Affiliated Hospital, Changsha Medical University, Changsha, 410219 China; 2 School of Information and Control Engineering, China University of Mining and Technology, Xuzhou, 221116 China; 3 College of Computer Science and Technology, Hunan Institute of Technology, Hengyang, 421002 China

## Abstract

MicroRNAs (miRNAs) act a significant role in multiple biological processes and their associations with the development of all kinds of complex diseases are much close. In the research area of biology, medicine, and bioinformatics, prediction of potential miRNA-disease associations (MDAs) on the base of a variety of heterogeneous biological datasets in a short time is an important subject. Therefore, we proposed the model of Composite Network based inference for MiRNA-Disease Association prediction (CNMDA) through applying random walk to a multi-level composite network constructed by heterogeneous dataset of disease, long noncoding RNA (lncRNA) and miRNA. The results showed that CNMDA achieved an AUC of 0.8547 in leave-one-out cross validation and an AUC of 0.8533+/−0.0009 in 5-fold cross validation. In addition, we employed CNMDA to infer novel miRNAs for kidney neoplasms, breast neoplasms and lung neoplasms on the base of HMDD v2.0. Also, we employed the approach for lung neoplasms on the base of HMDD v1.0 and for breast neoplasms that have no known related miRNAs. It was found that CNMDA could be seen as an applicable tool for potential MDAs prediction.

## Introduction

MicroRNAs (miRNAs) is a kind of short noncoding RNA (ncRNA) molecules with about 22 nucleotides in length which can regulate complementary messenger RNAs^1^. Unlike the miRNAs, long noncoding RNAs (lncRNAs) are a sort of heterogeneous ncRNAs with about 200 nucleotides and usually show less sequence conservation. Accumulating evidence indicates that miRNAs are participated in a wide variety of life process of cells, such as proliferation^2^, development^3^, aging^4^, viral infection^5^, metabolism^4,6^ and so on^5,7^. It is no surprise that miRNAs are closely related to a number of clinically important diseases^8,9^. For example, miR-335 and miR-126 were proved to be metastasis suppressor miRNAs in human breast cancer^10^. In addition, previous study also confirmed that the differential expression of miR-21, -31, -143 and -145 is closely participate in clinic pathologic features of colorectal cancer^11^. Therefore, identification of disease-related miRNAs would be beneficial for disease diagnosis, treatment, and prevention^12^. Currently, unlike traditional time-consuming biological experiments, adopting validation to the predicted miRNA-disease associations (MDAs) obtained from calculation models could reduce a lot of time and cost. Therefore, it is very significant to propose effective calculation models to infer potential MDAs^13–17^.

According to the idea that miRNAs with similar functions are usually relevant to similar diseases and the reverse is also true. some researchers built elaborate computational models for the identification of potential MDAs on the basis of known MDAs in databases only. For example, Li *et al*.^18^ developed a computational approach based on matrix completion, in which the adjacency matrix constructed from known MDAs was updated to gain final association scores of each miRNA-disease pair. Considering various types of known MDAs, Chen *et al*.^19^ constructed an restricted boltzmann machine (RBM) model to further predict four kinds of MDAs.

Based on the information of known MDAs and the corresponding similarity information of diseases and miRNAs, Chen *et al*.^20^ developed an effective method via combining all those information to construct a heterogeneous graph and then further inferred MDAs with the consideration of paths between miRNA nodes and disease nodes. Besides, this method could also be implemented to predict for new diseases (miRNAs). Through integrating the distribution information of *k* most similar neighbors per miRNA and the corresponding functional similarity between the miRNA and its neighbors, Xuan *et al*.^21^ proposed a reliable computational approach to infer novel MDAs. However, HDMP cannot predict disease-related miRNAs for new diseases. After computing miRNAs functional similarity (MFS), Xuan *et al*.^22^ proposed a prediction model via implementing random walk on constructed miRNA functional similarity network in which they assigned larger transition weights to marked nodes. At last, probability association scores of each disease-miRNA pair would be obtained and ranked. A calculation model was further built by Chen *et al*.^17^ in which miRNA’s *k*-nearest-neighbors (KNNs) and disease’s KNNs were respectively searched and then these KNNs would be ranked according to support vector machine. After that, they finally got all potential MDAs with weighted voting. Under the framework of semi-supervised learning, a novel model^23^ was presented for MDAs prediction via combining the optimal solutions in the miRNA space and disease space. Recently, Chen *et al*.^24^ proposed another prediction model through calculating within-score and between-score for both miRNAs and diseases which were then combined to obtain the final MDA scores.

Also, researchers put forward some other calculation approaches via considering relevant genes or proteins as a bridge to predict novel MDAs. For example, using a discrete probability distribution of hypergeometric, Jiang *et al*.^25^ presented a prediction model on the basis of the constructed integrated network. By connecting miRNAs to diseases with the proteins as a bridge between them, a calculation model was employed by Mork *et al*.^26^ through using a scoring scheme, which can greatly increase the model’s efficiency. Furthermore, Shi *et al*.^27^ implemented random walk on a built protein similarity network to identify MDAs.

By combining the known MDAs network and MFS network, a new calculating method was studied by Chen *et al*.^28^ by the analyzed of random walk with restart (RWR). It is worth noting that RWR is a very effective model for MDAs prediction. By adopting RWR, a novel model named Composite Network based inference for MiRNA-Disease Association prediction (CNMDA) was presented in the light of a multi-level network which was built by combination of Gaussian interaction profile kernel similarity (GIPKS) for lncRNAs, integrated similarity for miRNAs (ISMs) and diseases (ISDs), known MDAs, lncRNA-disease associations (LDAs) and miRNA-lncRNA interactions (MLIs). In addition, leave-one-out cross validation (LOOCV) and 5-fold cross validation were adopted in this paper to assess CNMDA’s effectiveness. It could be seen that the AUCs of LOOCV and 5-fold cross validation were respectively 0.8547 and 0.8533+/−0.0009. As for case studies, CNMDA was carried out on kidney neoplasms (KN), breast neoplasms (BN) and lung neoplasms (LN) to infer its associated miRNAs based on HMDD v2.0^29^. Also according to HMDD v2.0, we further infer novel miRNAs for BN after hiding its known associated miRNAs. At last, we carried out the case studies based on HMDD v1.0^30^ to infer LN-related miRNAs. Based on the above results, the effectiveness of CNMDA for MDAs prediction was validated.

## Results

### Cross validation

**Figure 1:**
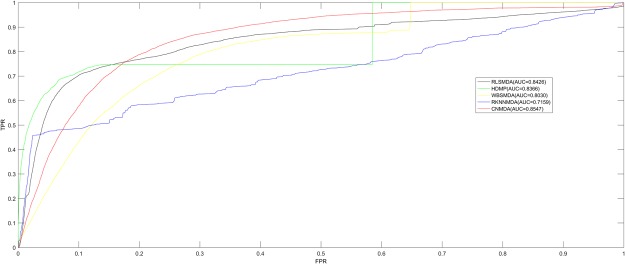
CNMDA got better AUCs of 0.8547 in the LOOCV in comparison of other four calculation approaches (RLSMDA, HDMP, WBSMDA, RKNNMDA).

In this paper, we carried out LOOCV and 5-fold cross validation to assess CNMDA’s prediction accuracy according to HMDD v2.0^29^ and then made comparison between CNMDA and four other classical computational models: RLSMDA^23^, HDMP^21^, WBSMDA^24^ and RKNNMDA^17^ (See Fig. 1). In LOOCV, test sample is one of the 5430 MDAs; training samples are the rest of 5429 known MDAs; candidate samples are those unlabeled 184155 miRNA-disease pairs. When each known MDA was taken to be the test sample, we would get association scores for all miRNA-disease pairs after implementing MCMDA and then the ranking of test sample among the candidate samples would be gained based on their association scores. We would say that the model makes a correctly prediction if the test sample ranked higher than the set threshold. Finally, we drew Receiver-Operating Characteristics (ROC) curve through computing the ratio of true positive rate to false positive rate. To evaluate CNMDA’s performance, we computed area under the ROC curve (AUC). If AUC = 1, CNMDA would possess perfect performance; If AUC = 0.5, CNMDA could only predict randomly. As a result, CNMDA, RLSMDA, HDMP, WBSMDA, RKNNMDA obtained AUCs of 0.8547 (0.8533+/−0.0009), 0.8426 (0.8569+/−0.0020), 0.8366 (0.8342+/−0.0010), 0.8030 (0.8185+/−0.0009) and 0.7159 (0.6723+/−0.0027) in the LOOCV (5-fold cross validation), respectively. Through comparative analysis with other method, the reliability and effectiveness of CNMDA for identification of potential MDAs were proved.

### Case studies

Three different case studies were also implemented to assess CNMDA’ performance. In the first case study, CNMDA was employed to predict KN-related miRNAs based on HMDD v2.0. Further, another two reliable MDA databases (dbDEMC and miR2Disease) would be utilized to validate the top 50 identified outcomes. In the second case study, we respectively inferred BN-associated miRNAs and BN-associated miRNAs after removing all known BN-associated miRNAs in HMDD v2.0. In the third kind of case studies, CNMDA was adopted to predict for LN according to associations in HMDD v1.0 and v2.0, respectively.

KN is a disease caused by cellular metabolic disorders^31^. If kidney tumors are detected and treated early and localized in the kidney, Patients would have a good disease-specific survival rate. Otherwise, patients have only an 18% two-year survival rate when they present with terminal disease^32^. With recent researches and studies, about two hundred and fifty thousand renal tumor patients are newly diagnosed annually, and KN’ morbidity and mortality continue to increase^33^. Many miRNAs related to KN have been found based on a large number of biological experiments. For example, in renal cell carcinoma (RCC), up regulation of miR-21 is related to kidney cancer that with lower survival rate^34^. Through targeting MMP-9 in RCC, miRNA-133b can suppress cell proliferation, migration and invasion^35^. Finally, we implemented CNMDA for potential KN-related miRNA prediction. It was found that 8 of the first 10 and 37 of the first 50 miRNAs were verified (See Supplementary Table 1). we also provided the whole scores of potential MDAs on the base of HMDD v2.0 (See Supplementary Table 2).

BN is a major chronic disease affecting adult women and detected breast neoplasms can be removed surgically^36^. However, if people with BN have not been detected, BN may develop into a life-threatening clinical recurrence in the next 5, 10, 15, or more years^37^. Recent experimental studies have provide evidences that miRNA-195 may work as latent biomarker for early BN detection^38^. To find the novel biomarkers for BN for the treatment of the disease is significant. In the second, we employed CNMDA for potential BN-related miRNA prediction. It was found that 5 of the first 10 and 31 of the first 50 miRNAs were verified (See Supplementary Table 3). Also, we implemented CNMDA for the prediction of BN by hiding all its confirmed associations in HMDD v2.0. This means that we would remove all known BN-associated miRNAs and predict potential BN-associated miRNAs based on other known associations and corresponding similarity information. Supplementary Table 4 presents the top 50 predicted outcomes and their verification evidences. As a result, 9 of the first 10 and 41 of the first 50 miRNAs were confirmed (See Supplementary Table 4).

LN is the primary reason of cancer deaths on a global scale^39^. The genetic and epigenetic damage caused by tobacco smoke is the main cause of the disease^40^. Obviously, it is urgent to find a more therapy systemic^39^. In squamous cell carcinoma, miR-126 have been verified to be down regulated and two miRNAs of miR-185∗, miR-125a-5p were up regulated^39^. MiR-205 were expressed differently in the non-small cell lung carcinoma (NSCLC)^40^. In order to test the stability of CNMDA, we employed CNMDA based on the associations in HMDD v2.0 and HMDD v1.0^30^, respectively. It was found that 20 and 28 of first 50 associated miRNAs for LN have been verified, respectively (See Supplementary Tables 5 and 6).

As seen in the results above, we can arrival at a conclusion that CNMDA possesses excellent predictive performance for the novel MDAs prediction.

## Discussions

As overwhelming evidences expounded that miRNAs are participated in all sorts of diseases. The development of new calculation approaches for predicting MDAs in a short time is important to further experimental validation. Accordingly, it is now possible to confirmed novel MDAs using biological experiments with low time and cost. Existing models are usually proposed based on four different calculation mechanisms^41^. Some scoring functions were constructed to prioritize disease-related miRNAs through carrying out probability distribution. Complex network algorithm-based prediction models were introduced through establishing complex network based on various data that are collected or calculated from different perspectives. Machine learning-based prediction models were introduced by using powerful machine learning algorithms. Moreover, multiple biological information-based models were put forward through constructing intermediate medium associations based on various biological datasets. We put forward the computing method of CNMDA to infer novel MDAs. In the model, we implemented RWR on a multi-level composite network that was built through combining collected and calculated data (ISD, ISM, GIPKS for lncRNAs, experimentally validated MDAs, MLIs and LDAs). From the evaluation results, it can be seen that the accuracy of our prediction model was superior in the comparison with other four models.

The main merits for the effective performance of CNMDA are as follows: Through taking advantage of multi-source information based on reliable database, it is no surprise that the integration strategy of CNMDA could predict potential MDAs effectively. Secondly, in comparison of local network information, RWR is an iterative process based on global network for the MDAs prediction. The attractive properties of global network information have been proved in the identification for potential disease-gene associations, MDAs^41,42^, LDAs^43^ and drug-target interaction^44^. Furthermore, CNMDA could identify novel diseases that have no known associated miRNAs. At last, the implementation of CNMDA only needs positive samples as training data. Since there is no known negative sample information, the forecasting precision of CNMDA is more convincing. However, some limitations also exist in the computation model of CNMDA. For example, the number of experimentally determined MDAs, LDAs and MLI is insufficient. For the number of known MDAs, only 5430 known MDAs were collected. The more the known MDAs, the higher forecasting precision the model. Importantly, the current forecasting precision still needs to be improved according to the evaluation of LOOCV.

## Methods

### MiRNA-disease associations

Experimentally confirmed MDAs used in this paper were come from high-quality database^29^. Through constructing a adjacency matrix *W*_*dm*_ to indicate the 5430 known MDAs, we made use of variables *nm* and *nd* to express the total amount of miRNAs and diseases in the known MDAs dataset, respectively.

1 $$\begin{array}{cc}{W}_{dm}(i,j) & =\,\{\begin{array}{ll}1, & if\,miRNA\,m(j)\,is\,related\,to\,disease\,d(i)\\ 0, & otherwise\end{array}\end{array}$$

### LncRNA-disease associations

The known LDAs was from the LncRNADisease^45^. After removing excess LDAs whose diseases don’t arise in the 5430 known MDAs mentioned above, we would acquire 250 known LDAs. Likewise, we built an adjacency matrix *W*_*dl*_(*i*,*j*) to indicate the 250 known LDAs. Variable *nl* refer to the number of lncRNAs in the 250 known LDAs.

2 $$\begin{array}{cc}{W}_{dl}(i,j) & =\,\{\begin{array}{ll}1, & if\,\mathrm{ln}\,cRNA\,l(j)\,is\,related\,to\,disease\,d(i)\\ 0, & otherwise\end{array}\end{array}$$

### MiRNA-lncRNA interactions

The known MLIs was from starBase v2.0^46^. In the same way, we need to delete excess MLIs whose miRNAs and lncRNAs do not exist in the 5430 known MDAs and 250 known LDAs. At last, 9088 known MLIs were gotten and an adjacency matrix *W*_*ml*_ was used to refer to the 9088 MLIs.

3 $$\begin{array}{cc}{W}_{ml}(i,j) & =\,\{\begin{array}{ll}1, & if\,\mathrm{ln}\,cRNA\,l(j)\,is\,related\,to\,miRNA\,m(i)\\ 0, & otherwise\end{array}\end{array}$$

### MiRNA functional similarity

The scores of MFS were obtained from http://www.cuilab.cn/files/images/cuilab/misim.zip^47^. We used *FS*(*i*,*j*) to indicate the score of MFS between miRNA *m*(*i*) and miRNA *m*(*j*).

### Disease semantic similarity model 1 (DSS1)

We put forward DSS1^48^ on the basis of Directed Acyclic Graph (DAG)^49^, which can be picked up according to MeSH descriptor of Category C. In the DAG = (*D*, *T*(*D*), *E*(*D*)) for disease *D*, all nodes are linked together from father to son using a straight line. The nodes of *D* and its elder can be collected into *T*(*D*) and *E*(*D*) referring to all the straight lines from father to son. Therefore, contribution of disease *d* in DAG(*D*) to the semantic value of disease *D* can be put forward.

4 $$\{\begin{array}{cclcc}{D}_{D}1(d) & = & 1 & if & d=D\\ {D}_{D}1(d) & = & \max \,\{{\rm{\Delta }}\ast {D}_{D}1(d\text{'})|d\text{'}\in children\,of\,d\} & if & d\ne D\end{array}$$

where Δ is the semantic contribution decay factor. It is worthy of being mentioned that the value of contribution for disease *D* to its own semantic value is 1. The semantic value of disease *D* could be put forward.

5 $$DV1(D)=\sum _{d\in T(D)}{D}_{D}1(d)$$

At last, DSS1 between *d*(*i*) and *d*(*j*) can be described.

6 $${\rm{SS1}}(d(i),d(j))=\frac{{\sum }_{t\in T(d(i))\cap T(d(j))}({D}_{d(i)}1(t)+{D}_{d(j)}1(t))}{DV1(d(i))+DV1(d(j))}$$

### Disease semantic similarity model 2 (DSS2)

In the DSS2^48^, due to the fact that a more specific disease *d* appearing in less DAGs would contribute more to the semantic value of disease *D*. Accordingly, the contribution made by *d* for the semantic value of *D* can be described by

7 $${D}_{D}2(d)=-\,\mathrm{log}[\frac{the\,number\,of\,DAGs\,including\,d}{the\,number\,of\,disease}]$$

DSS2 between disease *d*(*i*) and *d*(*j*) can be defined as follows:

8 $$DV2(D)=\sum _{d\in T(D)}{D}_{D}2(d)$$

9 $${\rm{SS2}}(d(i),d(j))=\frac{{\sum }_{t\in T(d(i))\cap T(d(j))}({D}_{d(i)}2(t)+{D}_{d(j)}2(t))}{DV2(d(i))+DV2(d(j))}$$

### Gaussian interaction profile kernel similarity

For disease *d*(*u*), we used *IP*(*d*(*u*)) to refer to row vectors of line *u* in *W*_*dm*_ on the basis of known MDA. Through watching whether *d*(*u*) is related to each miRNA, we computed GIPKS for diseases *d*(*u*) and *d*(*v*)^50^.

10 $$KD(d(u),\,d(v))=\exp (\,-\,{\gamma }_{d}{\Vert IP(d(u))-IP(d(v))\Vert }^{2})$$

where

11 $${\gamma }_{{\rm{d}}}={\gamma ^{\prime} }_{d}/(\frac{1}{nd}\sum _{u=1}^{nd}{\Vert IP(d(u))\Vert }^{2})$$

Similarly, GIPKS for miRNA *m*(*i*) and *m*(*j*) can be constructed.

12 $$KM(m(i),\,m(j))=\exp (\,-\,{\gamma }_{m}{\Vert IP(m(i))-IP(m(j))\Vert }^{2})$$

where

13 $${\gamma }_{{\rm{m}}}={\gamma }_{m}^{^{\prime} }/(\frac{1}{nm}\sum _{i=1}^{nm}{\Vert IP(m(i))\Vert }^{2})$$

For lncRNA *l*(*p*) and *l*(*q*), GIPKS between them can be constructed.

14 $$KL(l(p),\,l(q))=\exp (\,-\,{\gamma }_{l}{\Vert IP(l(p))-IP(l(q))\Vert }^{2})$$

15 $${\gamma }_{l}={\gamma }_{l}^{^{\prime} }/(\frac{1}{nl}\sum _{p=1}^{nl}{\Vert IP(l(p))\Vert }^{2})$$

### Integrated similarity for diseases (ISD) and miRNAs

We have taken into account combining GIPKS for diseases, DSS1 and DSS2 to compute ISD between diseases *d*(*u*) and *d*(*v*)^24^.

16 $$\begin{array}{cc}SD(d(u),d(v)) & =\,\{\begin{array}{ll}\frac{SS1(d(u),d(v))+SS2(d(u),d(v))}{2} & d(u)\,and\,d(v)\,has\,semantic\,similarity\\ KD(d(u),d(v)) & otherwise\end{array}\end{array}$$

Similarly, the ISM between miRNAs *m*(*i*) and *m*(*j*) can be put forward by the integration of GIPK for miRNA and MFS^24^.

17 $$\begin{array}{cc}{S}_{m}(m(i),m(j)) & =\,\{\begin{array}{ll}FS(m(i),m(j)) & m(i)\,and\,m(j)\,has\,functional\,similarity\\ KM(m(i),m(j)) & otherwise\end{array}\end{array}$$

### CNMDA

**Figure 2:**
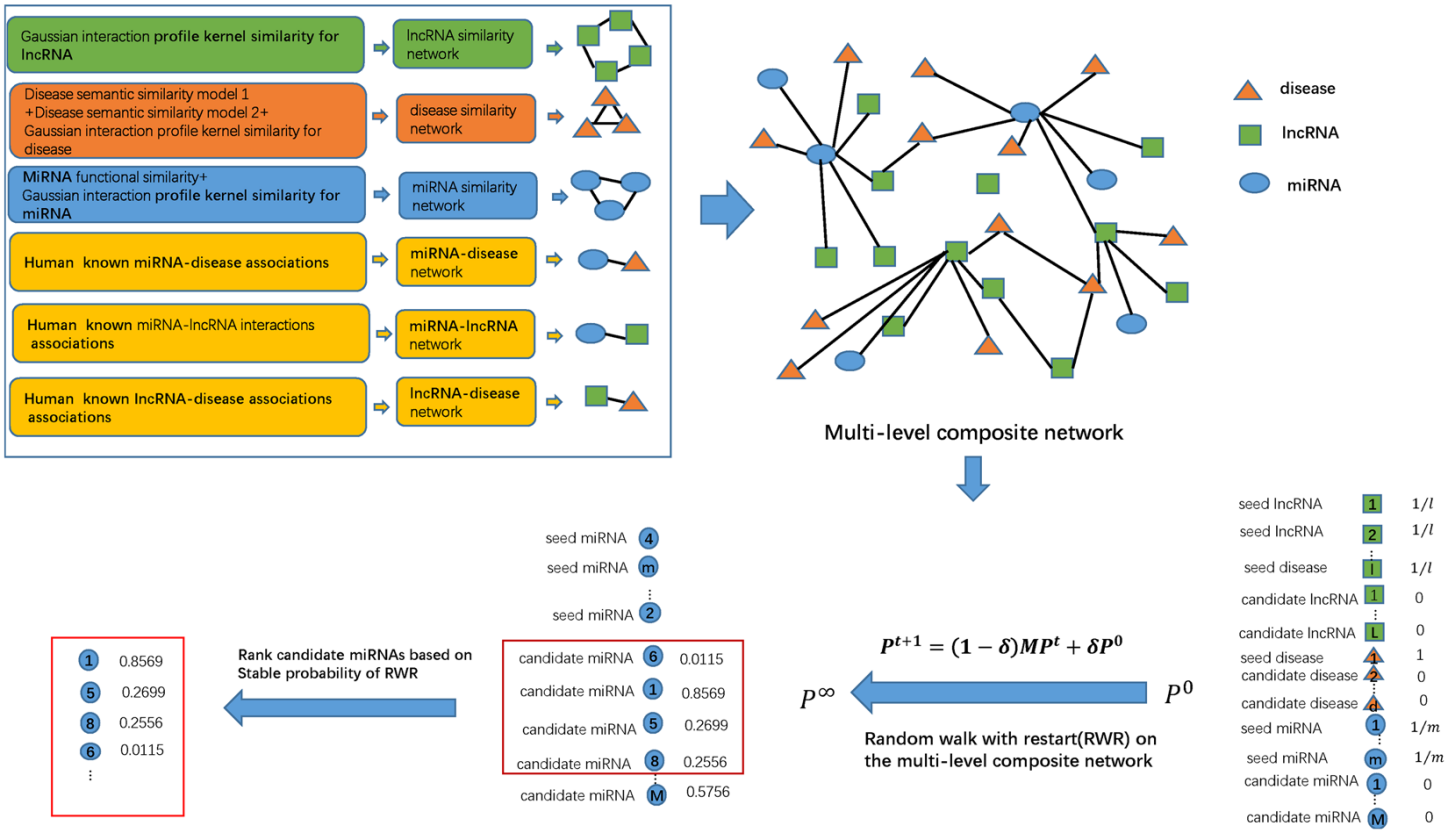
Flowchart of CNMDA for potential MDAs prediction in the light of HMDD v2.0. Each node in the constructed multi-level composite network possesses original probability ${p}^{0}$. Final scores ${p}^{\infty }$ for MDAs would be gotten after employing RWR.

Aiming at the prediction of potential MDAs, a computing method of CNMDA was stated. Carrying out RWR on a multi-level composite network that built by integration of ISM, ISD, GIPKS for lncRNA, known MDAs, LDAs and MLIs, final association scores of novel MDAs would be obtained (See Fig. 2, motivated by the studies of Yao *et al*.^51^). In our introduced model, we used ${W}_{l},{W}_{d},{W}_{m},{W}_{ld},{W}_{dm},{W}_{lm}$ to indicate the initial matrix of GIPKS for lncRNAs, ISD, ISM, known LDAs, known MDAs and known MLIs, respectively. Then, the initial matrix of the multi-level composite network can be defined as $$W=[\begin{array}{c}\begin{array}{ccc}{W}_{l} & {W}_{ld} & {W}_{lm}\end{array}\\ \begin{array}{ccc}{W}_{ld}^{T} & {W}_{d} & {W}_{dm}\end{array}\\ \begin{array}{ccc}{W}_{lm}^{T} & {W}_{dm}^{T} & {W}_{m}\end{array}\end{array}],$$ here, *T* refer to the transposition of matrix.

Global information based on the multi-level network would be captured through RWR algorithm. At each steps, seed nodes move to their immediate neighbors with a probability $(1-\delta )$ or go back to the seed nodes with a restart probability *δ*. ${P}^{0}$ was put forward to denote the original probability vector, and *P*^*t*+1^ was introduced to represent a probability vector of node at step *t* + 1, which could be described by:

18 $${P}^{t+1}=(1-\delta )M{P}^{t}+\delta {P}^{0}$$

where $\delta \in (0,1)$ is a restart probability. In the multi-level network, the initial seed node probability $${P}^{0}=[\begin{array}{c}\alpha \ast {u}_{0}\\ \beta \ast {v}_{0}\\ (1-\alpha -\beta )\ast {w}_{0}\end{array}],$$ where *α*, *β* and (1 − *α* − *β*) denote the weight of ISD network, ISM network and the network of GIPKS for lncRNAs, respectively. The corresponding *u*_0_, *v*_0_, *w*_0_ are the original probabilities of these three-similarity networks respectively. Here, *u*_0_ is calculated through assigning equal probability to all nodes in LDAs with a total to 1. Similarly, *v*_0_, *w*_0_ can be calculated.

Meanwhile, the transition matrix $$M=[\begin{array}{c}\begin{array}{ccc}{M}_{l} & {M}_{ld} & {M}_{lm}\end{array}\\ \begin{array}{ccc}{M}_{dl}^{T} & {M}_{d} & {M}_{dm}\end{array}\\ \begin{array}{ccc}{M}_{ml}^{T} & {M}_{md}^{T} & {M}_{m}\end{array}\end{array}]$$ can be computed in the light of adjacency matrix *W*. *M*(*i*,*j*) represents the transition probability from *i* to *j*. In the network of GIPKS for lncRNAs, the transition probability from lncRNA *i*(*l*_*i*_) to lncRNA *j*(*l*_*j*_) was put forward.

19 $$\begin{array}{c}{M}_{l}(i,j)={\rm{\Pr }}({l}_{j}|{l}_{i})\\ =\,\{\begin{array}{ll}(1-x-y){W}_{l}(i,j)/{\sum }_{j}{W}_{l}(i,j), & if\,\,{\sum }_{j}{W}_{ld}(i,j)\ne 0\,{\rm{and}}\,{\sum }_{j}{W}_{lm}(i,j)\ne 0\\ (1-x){W}_{l}(i,j)/{\sum }_{j}{W}_{l}(i,j), & if\,\,{\sum }_{j}{W}_{ld}(i,j)\ne 0\,{\rm{and}}\,{\sum }_{j}{W}_{lm}(i,j)=0\\ (1-y){W}_{l}(i,j)/{\sum }_{j}{W}_{l}(i,j), & if\,\,{\sum }_{j}{W}_{ld}(i,j)=0\,{\rm{and}}\,{\sum }_{j}{W}_{lm}(i,j)\ne 0\\ {W}_{l}(i,j)/{\sum }_{j}{W}_{l}(i,j), & if\,\,{\sum }_{j}{W}_{ld}(i,j)=0\,{\rm{and}}\,{\sum }_{j}{W}_{lm}(i,j)\ne 0\end{array}\end{array}$$

Similarly, in the ISD network, the transition probability from disease *i*(*d*_*i*_) to disease *j*(*d*_*j*_) was put forward.

20 $$\begin{array}{c}{M}_{d}(i,j)={\rm{\Pr }}({d}_{j}|{d}_{i})\\ =\,\{\begin{array}{ll}(1-x-z){W}_{d}(i,j)/{\sum }_{j}{W}_{d}(i,j), & if\,\,{\sum }_{j}{W}_{dm}(i,j)\ne 0\,{\rm{and}}\,{\sum }_{j}{W}_{ld}(j,i)\ne 0\\ (1-z){W}_{d}(i,j)/{\sum }_{j}{W}_{d}(i,j), & if\,\,{\sum }_{j}{W}_{dm}(i,j)\ne 0\,{\rm{and}}\,{\sum }_{j}{W}_{ld}(j,i)=0\\ (1-x){W}_{d}(i,j)/{\sum }_{j}{W}_{d}(i,j), & if\,\,{\sum }_{j}{W}_{dm}(i,j)=0\,{\rm{and}}\,{\sum }_{j}{W}_{ld}(j,i)\ne 0\\ {W}_{d}(i,j)/{\sum }_{j}{W}_{d}(i,j), & if\,\,{\sum }_{j}{W}_{dm}(i,j)=0\,{\rm{and}}\,{\sum }_{j}{W}_{ld}(j,i)=0\end{array}\end{array}$$

In the ISM network, the transition probability from miRNA $i({m}_{i})$ to miRNA $j({m}_{j})$ was put forward.

21 $$\begin{array}{c}{M}_{m}(i,j)={\rm{\Pr }}({m}_{j}|{m}_{i})\\ =\,\{\begin{array}{ll}(1-y-z){W}_{m}(i,j)/{\sum }_{j}{W}_{m}(i,j), & if\,\,{\sum }_{j}{W}_{dm}(j,i)\ne 0\,{\rm{and}}\,{\sum }_{j}{W}_{lm}(j,i)\ne 0\\ (1-y){W}_{m}(i,j)/{\sum }_{j}{W}_{m}(i,j), & if\,\,{\sum }_{j}{W}_{dm}(j,i)\ne 0\,{\rm{and}}\,{\sum }_{j}{W}_{lm}(j,i)=0\\ (1-z){W}_{m}(i,j)/{\sum }_{j}{W}_{m}(i,j), & if\,\,{\sum }_{j}{W}_{dm}(j,i)=0\,{\rm{and}}\,{\sum }_{j}{W}_{lm}(j,i)\ne 0\\ {W}_{m}(i,j)/{\sum }_{j}{W}_{m}(i,j), & if\,\,{\sum }_{j}{W}_{dm}(j,i)=0\,{\rm{and}}\,{\sum }_{j}{W}_{lm}(j,i)=0\end{array}\end{array}$$

In the LDAs network, the transition probability from lncRNA *i*(*l*_*i*_) to disease *j*(*d*_*j*_) was put forward.

22 $${M}_{ld}(i,j)={\rm{\Pr }}({d}_{j}|{l}_{i})=\{\begin{array}{ll}x{W}_{ld}(i,j)/{\sum }_{j}{W}_{ld}(i,j), & \,if\,{\sum }_{j}{W}_{ld}(i,j)\ne 0\\ 0, & {\rm{otherwise}}\end{array}$$

In the MLIs network, transition probability from lncRNA *i*(*l*_*i*_) to miRNA *j*(*m*_*j*_) was put forward.

23 $${M}_{lm}(i,j)={\rm{\Pr }}({m}_{j}|{l}_{i})=\{\begin{array}{ll}y{W}_{lm}(i,j)/{\sum }_{j}{W}_{lm}(i,j), & if\,{\sum }_{j}{W}_{lm}(i,j)\ne 0\\ 0, & {\rm{otherwise}}\end{array}$$

In the LDAs network, the transition probability from disease $i({d}_{i})$ to lncRNA $j({l}_{j})$ was put forward.

24 $${M}_{dl}(i,j)={\rm{\Pr }}({l}_{j}|{d}_{i})=\{\begin{array}{ll}x{W}_{ld}(j,i)/{\sum }_{j}{W}_{ld}(j,i), & if\,{\sum }_{j}{W}_{ld}(j,i)\ne 0\\ 0, & {\rm{otherwise}}\end{array}$$

In the MDAs network, the transition probability from disease $i({d}_{i})$ to miRNA $j({m}_{j})$ was put forward.

25 $${M}_{dm}(i,j)={\rm{\Pr }}({m}_{j}|{d}_{i})=\{\begin{array}{ll}z{W}_{dm}(i,j)/{\sum }_{j}{W}_{dm}(i,j), & if\,{\sum }_{j}{W}_{dm}(j,i)\ne 0\\ 0, & {\rm{otherwise}}\end{array}$$

In the MLIs network, the transition probability from miRNA $i({m}_{i})$ to lncRNA $j({l}_{j})$ was put forward.

26 $${M}_{ml}(i,j)={\rm{\Pr }}({l}_{j}|{m}_{i})=\{\begin{array}{ll}y{W}_{lm}(j,i)/{\sum }_{j}{W}_{lm}(j,i), & if\,{\sum }_{j}{W}_{lm}(j,i)\ne 0\\ 0, & {\rm{otherwise}}\end{array}$$

In the MDAs network, the transition probability from miRNA $i({m}_{i})$ to disease $j({d}_{j})$ was put forward.

27 $${M}_{md}(i,j)={\rm{\Pr }}({d}_{j}|{m}_{i})=\{\begin{array}{ll}z{W}_{md}(j,i)/{\sum }_{j}{W}_{md}(j,i), & if\,{\sum }_{j}{W}_{md}(j,i)\ne 0\\ 0, & {\rm{otherwise}}\end{array}$$

where $x,y,z$ are the jumping probability between the network of GIPKS for lncRNAs and ISD network, between the network of GIPKS for lncRNAs and ISM network, and between ISD network and ISM network, respectively. CNMDA is performed until the probabilities tend to a steady state, $${P}^{\infty }=[\begin{array}{c}\alpha \ast {u}_{\infty }\\ \beta \ast {v}_{\infty }\\ (1-\alpha -\beta )\ast {w}_{\infty }\end{array}]$$ (the range between *P*^*t*^ and ${P}^{0}$ computed by ${L}_{1}$ norm is smaller than 10^−6^). Then, the candidate miRNAs can be ranked according to ${w}_{\infty }$.

By incorporating MLIs and LDA into MDAs prediction, RWR was put forward on a constructed multi-level network to infer novel MDAs. In the network, because initial MLIs, LDAs and MDAs have more credibility, they all as weights in the RWR equations. Obviously, the one interaction and two associations play an equally important part in the network to disseminate information of miRNAs, diseases and lncRNAs for the novel MDAs prediction. In this study, we chose the same parameter as the one in previous literature^51^, which used RWR on the same multi-level composite network in their study. Therefore, we set the parameter $\delta$ to 0.7 and *x*, *y*, z, *α*, *β* to $\frac{1}{3}$.

## Electronic supplementary material

Supplementary material

Supplementary Table 1

Supplementary Table 2

Supplementary Table 3

Supplementary Table 4

Supplementary Table 5

Supplementary Table 6
