# Supplementary material for: Prediction of potential disease-associated microRNAs by composite network based inference
Source: Sci Rep. 2018 Oct 25;8:15813. doi: 10.1038/s41598-018-34180-6 (PMC6202421; doi:10.1038/s41598-018-34180-6)
Supplement: Supplementary file 1 — Supplementary material [file 41598_2018_34180_MOESM1_ESM.docx]

**Prediction of potential disease-associated microRNAs by composite network based inference**

Bin-Sheng He^1^, Jia Qu^2, *^, Min Chen^3, *^

^1^The First Affiliated Hospital, Changsha Medical University, Changsha, 410219, China

^2^School of Information and Control Engineering, China University of Mining and Technology, Xuzhou, 221116, China

^3^College of Computer Science and Technology, Hunan Institute of Technology, Hengyang, 421002, China

*Corresponding author

**Email**: [TB17060015B4@cumt.edu.cn](mailto:TB17060015B4@cumt.edu.cn); [chenmin@hnit.edu.cn](mailto:chenmin@hnit.edu.cn)

**Supplementary Information**

**Supplementary Table 1.** CNMDA was applied to predict the top 50 potential kidney neoplasms-associated miRNAs based on the dataset of known miRNA-disease associations that was downloaded from HMDD v2.0. The second and third column records top 1-50 related miRNAs and their association scores. The fourth column records the corresponding confirmed literature based on the database of dbDEMC and miR2Disease.

**Supplementary Table 2.** We applied CNMDA to prioritize all the candidate miRNA-disease pairs based on all the known MDAs in HMDD v2.0 database as training samples. This prediction result is released for further experimental validation and research.

**Supplementary Table 3.** CNMDA was applied to predict the top 50 potential breast neoplasms -associated miRNAs based on the dataset of known miRNA-disease associations that was downloaded from HMDD v2.0. T The second and third column records top 1-50 related miRNAs and their association scores. The fourth column records the corresponding confirmed literature based on the database of dbDEMC and miR2Disease.

**Supplementary Table 4.** CNMDA was applied to predict the top 50 miRNAs associated with breast neoplasms that without any known related miRNAs based on known associations that was downloaded from HMDD v2.0 database. The second and third column records top 1-50 related miRNAs and their association scores. The fourth column records the corresponding confirmed literature based on the database of HMDD v2.0, dbDEMC and miR2Disease.

**Supplementary Table 5.** CNMDA was applied to predict the top 50 potential lung neoplasms-associated miRNAs based on the dataset of known miRNA-disease associations that was downloaded from HMDD v2.0. The second and third column records top 1-50 related miRNAs and their association scores. The fourth column records the corresponding confirmed literature based on the database of dbDEMC and miR2Disease.

**Supplementary Table 6.** CNMDA was applied to predict the top 50 potential miRNAs associated with lung neoplasms based on the dataset of known miRNA-disease associations that was downloaded from HMDD v1.0 database. The second and third column records top 1-50 related miRNAs and their association scores. The fourth column records the corresponding confirmed literature based on the database of HMDD v2.0, dbDEMC and miR2Disease.
